# Water Sources and Their Protection from the Impact of Microbial Contamination in Rural Areas of Beijing, China

**DOI:** 10.3390/ijerph10030879

**Published:** 2013-03-05

**Authors:** Bixiong Ye, Linsheng Yang, Yonghua Li, Wuyi Wang, Hairong Li

**Affiliations:** 1 Institute of Geographical Sciences and Natural Resources Research, Chinese Academy of Sciences, Beijing 100101, China; E-Mails: bixiongye@126.com (B.Y.); yangls@igsnrr.ac.cn (L.Y.); wangwy@igsnrr.ac.cn (W.W.); lihr@igsnrr.ac.cn (H.L.); 2 Institute of Environmental Health and Related Product Safety, Beijing 100021, China

**Keywords:** drinking water, bacterial contamination, influencing factors

## Abstract

Bacterial contamination of drinking water is a major public health problem in rural China. To explore bacterial contamination in rural areas of Beijing and identify possible causes of bacteria in drinking water samples, water samples were collected from wells in ten rural districts of Beijing, China. Total bacterial count, total coliforms and *Escherichia coli* in drinking water were then determined and water source and wellhead protection were investigated. The bacterial contamination in drinking water was serious in areas north of Beijing, with the total bacterial count, total coliforms and *Escherichia coli* in some water samples reaching 88,000 CFU/mL, 1,600 MPN/100 mL and 1,600 MPN/100 mL, respectively. Water source types, well depth, whether the well was adequately sealed and housed, and whether wellhead is above or below ground were the main factors influencing bacterial contamination levels in drinking water. The bacterial contamination was serious in the water of shallow wells and wells that were not closed, had no well housing or had a wellhead below ground level. The contamination sources around wells, including village dry toilets and livestock farms, were well correlated with bacterial contamination. Total bacterial counts were affected by proximity to sewage ditches and polluting industries, however, proximity to landfills did not influence the microbial indicators.

## 1. Introduction

Bacterial contamination of drinking water is a major contributor to water-borne diseases such as diarrhea, nausea, gastroenteritis, typhoid dysentery and other health-related problems, especially in children and persons with weak immune systems [1,2]. Total bacterial count, total coliforms and *Escherichia coli* are common indicators of water contamination with disease causing pathogens used in China. Sources of total and fecal coliform in groundwater can include infiltration of domestic or wild animal fecal matter, effluent from leaking septic systems or sewage discharges and agricultural runoff [3]. According to the WHO standard for public drinking water, total coliforms and fecal coliforms in 100 mL of water must both be below detectable levels [4]. The Chinese drinking water quality guideline for total coliforms and *Escherichia coli* is also none detectable per 100 mL. The guideline for total bacteria count is less than 100 CFU·mL^−1^. In the rural areas around Beijing, there are 3,952 water wells which all are used for public water supply. There are approximately 3,590,000 rural inhabitants who depend on these wells to meet their daily water needs. Ground water accounts for 97.4% of total water sources. Because the parent geological material of the Beijing Plain consists of Quaternary unconsolidated sediments, contaminants can enter aquifers by infiltration of surface water through soil, sediments and rock or by surface runoff to the point where direct contamination occurs. The sanitary status of drinking water in rural areas is poor. Seventeen percent of wells are in the vicinity of contamination sources such as sewage ditches (carrying wastewater from homes, businesses, and industries), landfills, village dry toilets, livestock farms and polluting industries (casting factories, chemical factories, food processing plants, *etc.*). Drinking water in many of China’s rural areas is unhealthy, with 44.36% failing to meet government standards [5]. Most people living in rural areas do not have their drinking water disinfected, and bacterial contamination has been regarded as the greatest potential problem impacting drinking water [5]. Drinking water is commonly collected from its source area and transferred to water treatment plants for chlorination and then sent into the distribution system in rural areas. Only in a few large water treatment plants, the drinking water treatment processes includes setting, filtration and chlorination. It is well recognized that failure to protect water sources and inadequate water treatment are the primary reasons for drinking water contamination with bacteria [6]. Nevertheless, few quantitative studies of the effects of water sources and their protection on bacterial contamination in China have been carried out [7].

Beijing, the capital of China, is located in the Haihe River basin. It has a semi-arid and semi-humid continental monsoon climate, which leads to its low precipitation. Ground water is the main raw water supply for Beijing rural areas. It has been estimated that if bacterial contamination were solved, the Beijing rural drinking water compliance rate would reach 91.3%, and 25% of the risk associated with waterborne infectious diseases could be eliminated [8]. Therefore, in order to investigate the bacterial contamination of drinking water and prevent the occurrence of water-borne disease in developing countries, this study was conducted to analyze drinking water quality with respect to bacterial contamination in rural areas of Beijing, and to identify possible causes of bacteria in drinking water samples that are related to water sources and their protection.

## 2. Experimental Section

**Figure 1:**
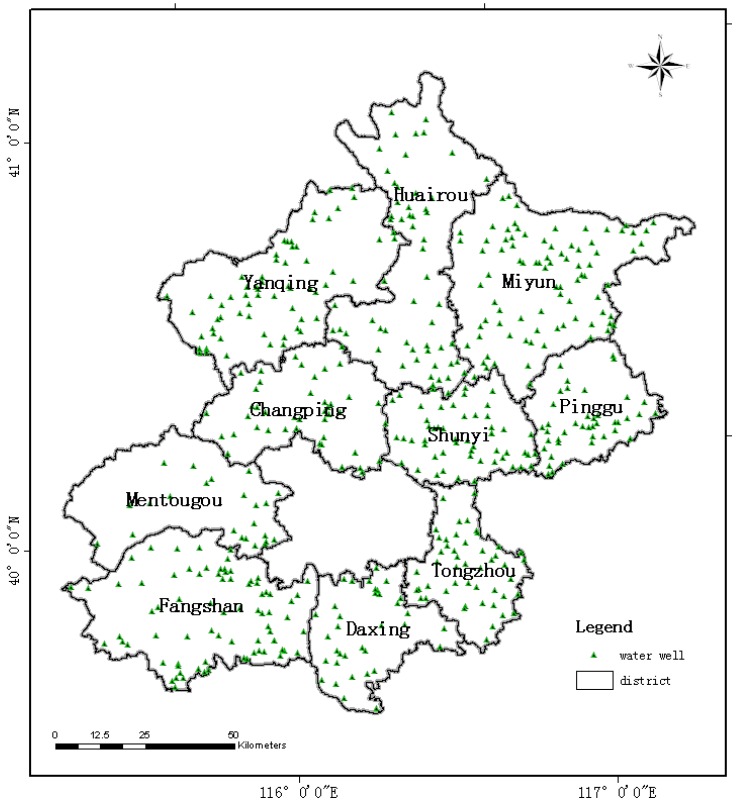
Map of sample sites.

This study was conducted in 10 rural districts of Beijing, China. A map of the sample sites is shown in Figure 1. Nearly 13% of the water treatment plants and 17% of the total population in these rural areas were surveyed and 503 water treatment plants were sampled once in summer and once in winter. For all water treatment plants, two samples (raw water and finished water) were selected for the purposes of microbial analysis. A total of 2,012 samples were collected from all water treatment plants. Microbial analysis of water was conducted to determine the total bacterial count, total coliforms and *Escherichia coli*. The standard plate counting method was used to enumerate the total bacterial count [9]. The total coliforms and in water were analyzed using the multiple tube most probable number (MPN) fermentation technique utilizing enzymes β-D-galactosidase and β-D-gluguronidase [10].

Some parameters of water sources and their protections including water source type (ground water = 1, surface water (including spring water) = 2), well depth, well cap (sealed = 1, not sealed = 2), wellhead above/below ground level (above = 1, below = 2), well housing (housing = 1, no housing = 2) and contamination sources (sewage ditches, landfills, village dry toilets, livestock farms and polluting industries) around water sources (contamination source = 1, no contamination source = 2) were investigated and quantified. Spearman-correlation analysis and paired-samples *t* test were used to explore relationships between bacterial contamination and well depth. Mann-Whitney U-test was used for testing the differences between the explanatory groups (water source, wellhead protection, pollution sources, *etc.*).

## 3. Results and Discussion

### 3.1. Bacterial Contamination in Drinking Water

**Figure 2:**
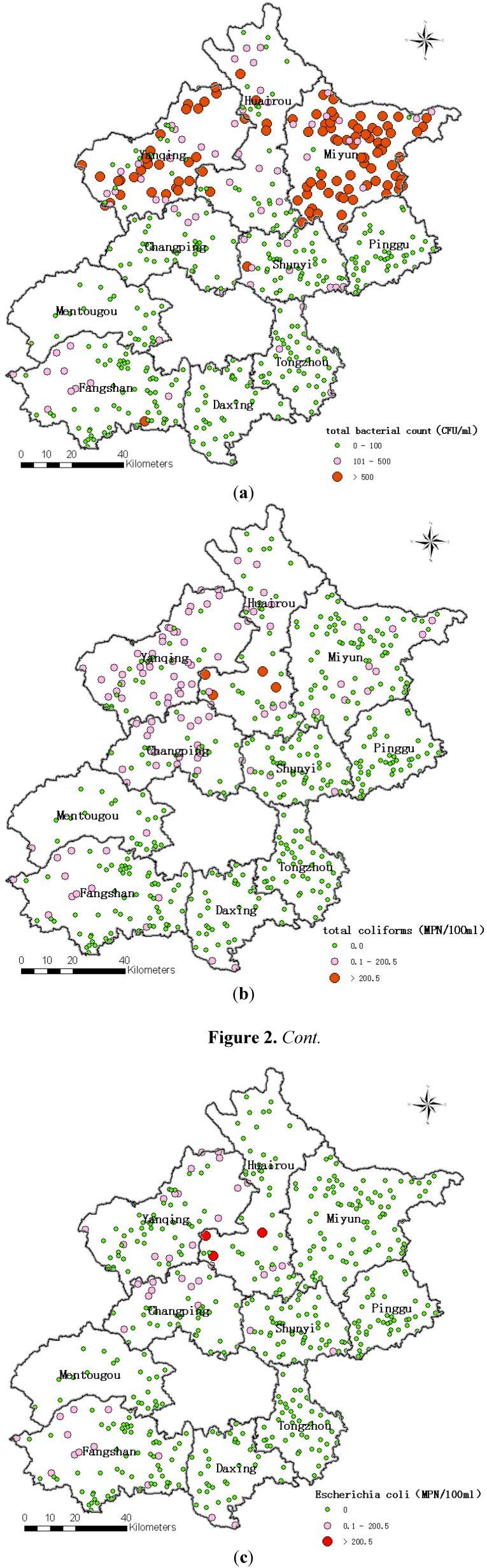
Bacterial contamination in finished water in rural areas around Beijing in summer by districts. (**a**) total bacterial count; (**b**) total coliforms; (**c**) *Escherichia coli*; N = 503.

The total bacterial count in water samples ranged from non-detectable to 88,000 CFU/mL (Table 1). As shown in Figure 2(a), in water from rural Beijing, the total bacterial count was higher in the north than the south. The percentage of samples with total bacteria count higher than 100 CFU/mL was highest in the Miyun district, followed by the Yanqing and Huairou districts. The concentration of total coliforms ranged from non-detectable to 1,600 MPN/100 mL (Table 1). As shown in Figure 2(b), in water in rural areas around Beijing, the total coliforms were also higher in the north than the south. The total coliforms at some sampling sites in the districts of Yanqing, Changping, Huairou and Fangshan exceeded the WHO standard for public drinking water that total coliform bacteria must not be detectable in any 100 mL sample [11]. The concentration of *Escherichia coli* ranged from non- detectable to 1,600 MPN/100 mL (Table 1), and as shown in Figure 2(c), *Escherichia coli* levels were higher in the north than the south. *Escherichia coli* contamination was more serious in the Yanqing, Changping and Huairou districts than the other districts investigated in this study.

### 3.2. Water Source and Wellhead Protection

Water source type, well depth, well cap, wellhead above/below ground level and well housing were selected to explore bacterial contamination caused by water sources and their protection. The results of Spearman-correlation analysis, paired-samples *t* test and Mann-Whitney U-test of the water sources or their protection and bacterial contamination are shown in Table 2. The same trends were observed among the three microbial indicators (total bacterial count, total coliforms and *Escherichia coli*) and water source or its protection. These results demonstrate that water source, well depth and wellhead protection could affect bacterial contamination.

**Table 1:** Descriptive statistics of bacterial contamination in drinking water.

		TBC (CFU/mL)	TC (MPN/100 mL)	*E. Coli* (MPN/100 mL)			TBC (CFU/mL)	TC (MPN/100 mL)	*E. Coli* (MPN/100 mL)
Changping	N	172	172	172	Miyun	N	340	340	340
	median	2	ND	ND		median	1,100	ND	ND
	minimum	ND	ND	ND		minimum	8	ND	ND
	maximum	370	201	45		maximum	4,200	23	ND
Daxing	N	140	140	140	Pinggu	N	162	162	162
	median	2	ND	ND		median	ND	ND	ND
	minimum	ND	ND	ND		minimum	ND	ND	ND
	maximum	100	ND	ND		maximum	350	23	23
Fangshan	N	298	298	297	Shunyi	N	208	208	208
	median	ND	ND	ND		median	6	ND	ND
	minimum	ND	ND	ND		minimum	ND	ND	ND
	maximum	800	33	33		maximum	2,900	8	8
Huairou	N	207	207	207	Tongzhou	N	188	188	188
	median	16	ND	ND		median	2	ND	ND
	minimum	1	ND	ND		minimum	ND	ND	ND
	maximum	2,600	1,600	1,600		maximum	370	8	8
Mentougou	N	92	92	92	Yanqing	N	248	248	245
	median	3	ND	ND		median	165	3	ND
	minimum	ND	ND	ND		minimum	ND	ND	ND
	maximum	150	49	ND		maximum	88,000	1,600	201

N: the number of samples; ND: no detectable; TBC: total bacterial count; TC: total coliform; *E. coli*: *Escherichia coli*.

**Figure 3:**
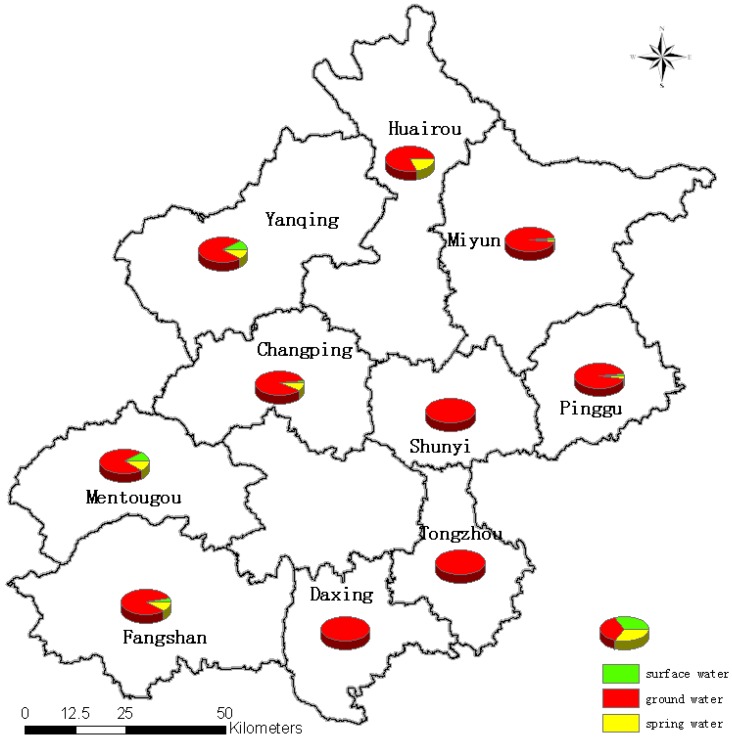
Distribution of water source types in rural areas around Beijing.

Minimization of contamination of a water system begins with selection of the system itself, and especially selection of the water source [12,13,14,15]. As shown in Table 2, Mann-Whitney U test demonstrates that water sources has influenced the bacterial contamination significantly (*p* < 0.01). Bacterial contamination was common in water treatment plants for which surface water or spring water was the source. The distribution of water source types of rural areas of Beijing is shown in Figure 3. Surface water and spring water were mainly found in west, north-west and south-west of Beijing (Yanqing, Huairou, Changping, Mentougou and Fangshan districts), and they could be more easily be contaminated by microorganisms than ground water which may be a cause for explaining the relatively serious bacterial contamination of drinking water north of Beijing.

**Table 2:** Mann-Whitney U test, paired-samples t test and Spearman-correlation analysis of water source types and well protection.

		TBC			TC			*E. Coli*		
	N	Sum of Ranks	Z	*p*	Sum of Ranks	Z	*p*	Sum of Ranks	Z	*p*
Well seal	1,614	1,388,953.5	−8.69	0.00	1,423,757.0	−4.95	0.00	1,435,982.5	−2.52	0.01
Well unseal	175	212,201.5			177,398.0			161,595.5		
Wellhead above ground level	1,172	793,316.0	−3.15	0.00	792,041.5	−5.47	0.00	804,776.5	−2.33	0.02
Wellhead below ground level	209	160,955.0			162,229.5			148,113.5		
Well housing	1,122	703,174.0	−12.95	0.00	754,351.0	−6.57	0.00	772,928.5	−1.92	0.05
No well housing	264	258,017.0			206,840.0			186,876.5		
Surface water	184	215,173.5	−5.43	0.00	236,630.5	−12.45	0.00	220,220.5	−14.88	0.00
Ground water	1,737	1,630,907.5			1,609,450.5			1,616,265.5		
Well depth	1,693	-	6.22 (*t*)	0.00	-	−30.07 (*t*)	0.00	-	−50.72 (*t*)	0.00
	1,693	-	−0.15 (*r*)	0.00	-	−0.34 (*r*)	0.00	-	−0.11 (*r*)	0.02

TBC: total bacterial count; TC: total coliform; *E. coli*: *Escherichia coli;* N: the number of samples; Z: Z value of Mann-Whitney U test; *p* sig. (2-tailed); *r*: correlation coefficient of Spearman-correlation analysis; *t* value of paired-samples *t* test.

Wellhead protection is another important factor influencing bacterial contamination [15]. Contaminated surface water can enter a well if the wellhead does not extend high enough above the ground surface, the well cap has been broken or removed or the well has no well housing. As shown in Table 2, Mann-Whitney U test demonstrates that well sealed/unsealed, wellhead above/below ground and well housing/no well housing influenced the microbial indicators significantly. Moreover, the bacterial contamination was serious in the water of wells that were not closed, had no well housing or had a wellhead below ground level. As shown in Table 3, the proportion of well sealed, well heads above ground and well housing was high in Changping, Daxing, Pinggu, Shunyi and Tongzhou districts, and the level of well sealing reached 87% in the Mentougou district. The wellhead protection was good and bacterial contamination was relatively low in these areas. However, the proportion of well seals in Miyun, Yanqing, Huairou and Fangshan districts was no larger than 75%, the proportion of above ground wellheads in Miyun, Yanqing, Huairou, Mentougou and Fangshan districts was less than 70% and the proportion of well housings in Miyun, Yanqing, Huairou, Mentougou and Fangshan districts was no larger than 52%, indicating that wellhead protection was bad and bacterial contamination may be serious in these districts.

**Table 3:** Wellhead protections account for the proportion of the total number of survey.

District	The number of samples	Well seal	Wellhead above ground	Well housing
Changping	43	93%	93%	93%
Daxing	37	83%	83%	83%
Fangshan	80	75%	68%	20%
Huairou	58	73%	29%	51%
Mentougou	23	87%	48%	52%
Miyun	85	65%	69%	44%
Pinggu	41	90%	87%	90%
Shunyi	54	98%	98%	98%
Tongzhou	51	98%	98%	98%
Yanqing	63	72%	3%	8%

### 3.3. Contamination Sources around Water Sources

The contamination sources around wells also affected bacterial contamination of water. Contamination sources such as sewage ditches, landfills, village dry toilets, livestock farms and polluting industries within 30 m of the water source were investigated. As showed in Table 4, Mann-Whitney U test demonstrates that sewage ditches and polluting industries could influence the total bacterial count significantly (all *p* < 0.01).

**Table 4:** Mann-Whitney U test of the contamination sources around water sources.

		TBC			TC			*E. Coli*		
	N	sum of ranks	Z	*p*	sum of ranks	Z	*p*	sum of ranks	Z	*p*
Sewage ditch	34	5,326.0	−0.27	0.79	7,022.5	−3.24	0.00	5,373.0	−0.08	0.94
No sewage ditch	283	45,077.0			43,380.5			44,713.0		
Landfills	31	3,835.5	−0.73	0.47	4,131.5	−0.39	0.70	3,892.0	−0.60	0.55
No landfills	225	29,060.5			28,764.5			29,004.0		
Village dry toilet	420	103,948.5	−2.23	0.02	101,810.5	−2.62	0.01	103,990.0	−1.91	0.05
No village dry toilet	78	20,302.5			22,440.5			20,261.0		
Livestock farm	17	2,028.0	−1.15	0.18	2,668.5	−2.46	0.01	2,008.0	−1.02	0.19
No livestock farm	218	25,702.0			25,061.5			25,722.0		
Polluting industries	33	4,396.5	−0.40	0.69	3,311.5	−2.81	0.00	4,257.0	−1.43	0.15
No polluting industries	238	32,459.5			33,544.5			32,599.0		

TBC: total bacterial count; TC: total coliform; *E. coli*: *Escherichia coli*; N: the number of samples; Z: Z value; *p* sig. (2-tailed).

The effluent from sewage ditches and polluting industries contained some chemical substances which could change the water pH value, and inhibit aquatic microbial growth [16]. The Mann-Whitney U test demonstrates that village dry toilets influenced total coliforms (*p* = 0.02), total bacterial count (*p* = 0.01) and *Escherichia coli* (*p* = 0.05) significantly. Livestock farms could influence total bacterial count significantly (*p* = 0.01), but influence total coliforms and *Escherichia coli* moderately (*p* = 0.18 and 0.19). The coliforms bacterial group, including total coliforms and *Escherichia coli*, may occur in water due to faecal contamination, *i.e.*, discharge of faeces by humans and other animals in water [17,18,19]. The waste from village dry toilets and livestock farms was arbitrarily discharged, did not pass through pipelines or ditches, and was not subject to sewage treatment. Therefore, village dry toilets could strongly influence the bacterial contamination and livestock farms could increase bacterial contamination. Landfill leachate contains a variety of pollutants that may potentially contaminate ground water and affect the quality of surface waters and well waters [20,21], however, no significant differences were observed in the three microbial indicators (*p* = 0.47, 0.70 and 0.55), and landfills did not influence these indicators. Because of environmental regulations, most landfill sites in rural areas are far from water sources, and landfill leachate is controlled, which may explain this lack of correlation.

## 4. Conclusions

The total bacterial count, total coliforms and *Escherichia coli* in water samples ranged from non-detectable to 88,000 CFU/mL, non-detectable to 1,600 MPN/100 mL and non-detectable to 1,600 MPN/100 mL, respectively. Bacterial contamination mainly occurred in water plants whose water source was surface water or spring water. Shallow groundwater and poorly sealed wellheads were the major factors that caused drinking water bacterial contamination in rural areas. Moreover, the absence of well housings and wellheads below ground level were the primary reasons for serious bacterial contamination in drinking water.

In the rural areas of Beijing, areas of serious bacterial contamination of drinking water were mainly located north of Beijing in the Miyun, Yanqing, Huairou and Changping districts. Shallow water and poor wellhead protection explained the bacterial contamination in the Miyun and Huairou districts. No well housing and wellheads below ground level were the main reasons for bacterial contamination of the Yanqing District. The well depth and wellhead protection in the Changping district showed no obvious problems, indicating that bacterial contamination may be occurring during the water treatment processes. Moreover, because many village dry toilets were located around the water sources, there were indicators of bacterial contamination found in water samples of Shunyi and Fangshan district.
